# Undergraduate students’ perception of cardiorespiratory physiology during exercise: teleological vs. mechanistic thinking

**DOI:** 10.1186/s12909-024-05073-2

**Published:** 2024-01-29

**Authors:** Marcela S. Araújo, André L. Teixeira, Amanda Esteves, Jeann Sabino-Carvalho, Lauro C. Vianna

**Affiliations:** 1 NeuroV̇ASQ̇ - Integrative Physiology Laboratory, Faculty of Physical Education, University of Brasília, Brasília, DF Brazil; 2 Department of Human Health and Nutritional Sciences, University of Guelph, Guelph, ON Canada; 3 Division of Renal Medicine, Department of Medicine, Emory University School of Medicine, Atlanta, GA USA

**Keywords:** Exercise physiology, Students, Teleological thinking, Physiology education, Teaching

## Abstract

**Background:**

Physiology is widely recognized as a difficult course, which can potentially increase students’ withdrawal and failures rates. Several factors are likely contributing to the difficulties in learning physiology, including inherent features of the discipline as well as aspects related to instructions and/or students’ perception. With regards to the later, it is currently unknown how students of exercise physiology think and explain physiology in terms of its cause or consequence (i.e., teleological or mechanistic thinking). Therefore, the aims of the present study were to determine 1) whether undergraduate students’ perception of cardiorespiratory physiology during exercise follows a predominant teleological or mechanistic thinking, and 2) whether prior enrollment in physiology courses can influence the predominance of teleological vs. mechanistic thinking.

**Methods:**

The test instrument was an online questionnaire about exercise physiology consisting of nine incomplete sentences about exercise physiology where students had to choose between a teleological or a mechanistic complement. The questionnaire was administered to undergraduate students in the following areas: 1) Movement Sciences (*n* = 152), 2) Health-related (*n* = 81) and, 3) Health-unrelated programs (*n* = 64). Students in Movement Sciences and Health-related programs were also analyzed separately in the following categories: 1) students who previously undertook physiology courses, and 2) students who did not take physiology courses.

**Results:**

Overall, all groups presented a percentage of teleological thinking above 58%, which is considerably high. Teleological thinking was significantly higher in health-unrelated programs than health-related and movement sciences programs (76 ± 16% vs. 58 ± 26% vs. 61 ± 25%; *P* < 0.01). Further, students with prior enrollment in physiology classes presented a significantly lower percentage of teleological thinking than students without physiology classes (59 ± 25% vs. 72 ± 22%, respectively; *P* < 0.01), but the overall teleological reasoning remained predominant.

**Conclusions:**

These results confirm the hypothesis that undergraduate students tend to present teleological as opposed to mechanistic thinking in exercise physiology. Furthermore, although undergraduate students with prior enrollment in physiology classes presented significantly lower teleological thinking, it remained highly predominant suggesting that teleological thinking is partially independent of the degree of familiarity with this discipline.

## Background

Exercise physiology is an essential course to a more comprehensive understanding of the effects of exercise on human physiology. However, physiology is widely perceived as a difficult course, which can potentially increase students’ withdrawal and failure rates [1, 2]. Several factors may be contributing to the difficulties in learning physiology, including inherent features of the discipline as well as aspects related to instructions and/or students’ perception [1]. With regard to the latter, an overview of definitions of physiology given by the main physiological societies, shows that the concept of function is central in physiology [3]. However, the concept of function in science is highly controversial due to its teleological (i.e., goals-directed) component [3–6]. For example, the rationale that “we breathe because we need oxygen” focuses the existence of respiratory processes on the consequence (or the function of a biological phenomenon) while disregarding its mechanism (i.e., inherent activity of medullary neurons and peripheral chemoreceptors). Although explanations of biological phenomena are a fundamental element of the science teaching and learning [4], it is currently unknown how students of exercise physiology think and explain physiology in terms of its cause or consequence (i.e., mechanistic or teleological thinking).

Children and adults have a teleological tendency to attribute functions to biological phenomena [7, 8]. In an attempt to better understand students’ tendency to teleological reasoning about general physiology, Richardson [9] presented 10 biological phenomena from the human body to students in elementary and advanced-level physiology courses. Students chose between a mechanistic and a teleological explanation which was perceived to best explain the biological phenomena. The results demonstrated that the majority of high school and undergraduate students preferred teleological instead of mechanistic explanations. Although both approaches are important, understanding students' notions about physiological responses to exercise is essential to prevent students’ misconceptions when thinking about physiology [10, 11]. Of note, these misconceptions are characterized by a difficulty in distinguishing between the “end result”, a physiological response, and the cause that leads to the “end result”. Additionally, teleological and mechanistic explanations are complementary and fundamental to the education process, however, the overgeneralized application of teleological thinking leads to the development of an incomplete understanding of natural phenomena, misconceptions, and the limitation of scientific knowledge [4, 8, 12]. Nevertheless, students' tendency to teleological or mechanistic thinking in exercise physiology remains an open area of investigation. In addition, whether previous enrollment in programs related to physiology can influence the degree of students’ teleological or mechanistic perception remains unknown.

Given this background, herein, we aimed to investigate 1) whether undergraduate students’ perception of cardiorespiratory physiology during exercise follows a predominant teleological or mechanistic thinking and 2) whether prior enrollment in physiology courses can influence the predominance of teleological vs. mechanistic thinking. We hypothesized that undergraduate students have a predominant teleological reasoning about exercise physiology, and this would be lower in students previously enrolled in physiology courses. If confirmed, these findings will support the concept that teleological thinking can be dependent on the degree of familiarity with physiology courses.

## Methods

### Ethical approval

Participants in the present study included groups of undergraduate students from different departments of a public university. All study procedures were approved by the institution’s Research Ethics Committee in accordance with the Declaration of Helsinki. Written informed consent was obtained from all participants prior to the completion of the current study. All students were told that their participation was voluntary and that they could unconditionally withdraw at any time. Each participant read and agreed with a specific informed consent form before the participation. After agreeing, they received the informed consent via email.

### Students

The present study was conducted in groups of undergraduate students from different faculties (i.e., different instructors) and departments of a public university. Based on student undergraduate enrollment, participants were grouped into one of the following undergraduate programs: 1) Movement Sciences (*n* = 152), 2) Health-related programs (*n* = 81), and 3) Health-unrelated programs (*n* = 64). Students in both movement sciences and health-related programs have physiology in their academic curriculum. However, students in movement sciences have exercise physiology courses specifically, while students in health-related programs have more diverse and general physiology courses. Students from health-unrelated programs did not have any human physiology-related courses or content in their academic curriculum.

Participants in Movements Sciences and Health-related programs also presented with a range of prior education in physiology, ranging from no prior prerequisite physiology courses, to completion of one or more prerequisite physiology courses. Therefore, students in Movement Sciences and Health-related programs were analyzed separately in the following categories: 1) students who previously had taken physiology courses, and 2) students who had never been enrolled in physiology courses. All students from health-unrelated programs had no physiology courses and as a result, this group was not analyzed separately.

### Test instrument

To gather students’ perceptions of exercise physiology, the test instrument for this study was an online questionnaire consisting of nine statements based on items used by Richardson [9], but focused on exercise physiology (Table 1). Each statement consisted of an incomplete sentence about a physiological response to exercise and was evaluated and revised by two experts in the field (A.L.T and L.C.V.) before data collection in order to guarantee discriminative power between the answers in each question. The discriminative power was verified in a pilot study involving 32 sport sciences students. In this pilot study a lecture on teleological vs. mechanistic approaches about body function was given after the completion of the questionnaires. Following this, students had the opportunity to answer the questionnaires once again and the average teleological response was only 17%, highlighting the discriminative power of our test instrument.

**Table 1:** Statements in test instrument

1- During physical activity, oxygen enters the skeletal muscle tissue from the blood because: a) Oxygen content inside muscle tissue decreases as the oxygen is used b) Muscle require oxygen to produce energy
2- During exercise, heart rate increases: a) Due to the need for oxygen-rich blood for active tissues b) Due to vagal withdraw and progressive stimulation of sympathetic activity
3- The gas exchange in the lungs occurs: a) Due to the pressure gradient of oxygen and carbon dioxide between the alveoli and blood capillaries b) Due to the need to supply oxygen to the tissues and eliminate carbon dioxide on exhalation
4- Increased oxygen transport in the blood occurs: a) Due to the need for oxygen supply to produce ATP and assist in muscle contraction b) Due to the increase in bonds between oxygen and hemoglobin, which is essential for the functioning of tissues
5- During exercise, blood pressure does not decrease because: a) Muscle afferences send information to the central nervous system, which inhibits the action of baroreflex and modulates autonomic nervous activity b) There is an increase in heart rate and blood flow to match delivery of oxygen and nutrients of the contracting skeletal muscles metabolic demand
6- The respiratory cycle is regulated by: a) The need to adapt the respiratory rate to the metabolic demand b) Inherent activity of medullary neurons and peripheral chemoreceptors
7- During exercise, the increase in pulmonary ventilation is regulated by: a) Interactions between cortical and peripheral feedback that stimulate respiratory neurons in the medullary respiratory neurons b) Increase in tidal volume and respiratory rate to match the metabolic demand of the active muscle of oxygen input and carbon dioxide output
8- During exercise, blood flow is regulated: a) To redistribute oxygen-rich blood to active muscles and match metabolic demand b) By the interaction between sympathetic nerve activity, vasoconstriction, peripheral receptors and local mechanisms of vasodilatation
9- Sweating occurs whenever: a) The body needs to eliminate excess heat b) The muscle surround the sweat glands contract

Teleological answers: 1; b—2; a—3; b—4; a—5; b—6; a—7; b—8; a—9; a

For the same physiology phenomenon, students had to select one of two possible choices – a teleological or a mechanistic – that they felt best completed the statement. The students were told that there was no wrong answer, as long as they chose according to what they thought. It was emphasized that the intention was not to evaluate them, but to verify their perceptions about the functioning of the human body during exercise. The students were not informed that the choices had different ideologies.

### Test procedures

The test instrument was widely publicized on social networks and communication vehicles for undergraduate students. Students had access to the online questionnaire through an invitation letter, where they were asked to participate in academic research. The questionnaire was available after the participants read and agreed with the informed consent form. They were instructed to answer only once. There was no time limit to choose sentences.

### Statistical analysis

Shapiro–Wilk’s normality test was used to verify the normal distribution of the data. Statistical comparisons of the percentage of teleological thinking between-groups were made by one-way ANOVA. The Bonferroni’s post hoc test was used when significant F values were found. The significance level was set at *P* ≤ 0.05. All data are presented as mean ± standard deviation (SD), unless otherwise stated. All analyses were performed using Statistical Package for the Social Sciences (IBM Corp. Released 2011. IBM SPSS Statistics for Windows, Version 20.0. Armonk, NY: IBM Corp.).

## Results

**Fig. 1:**
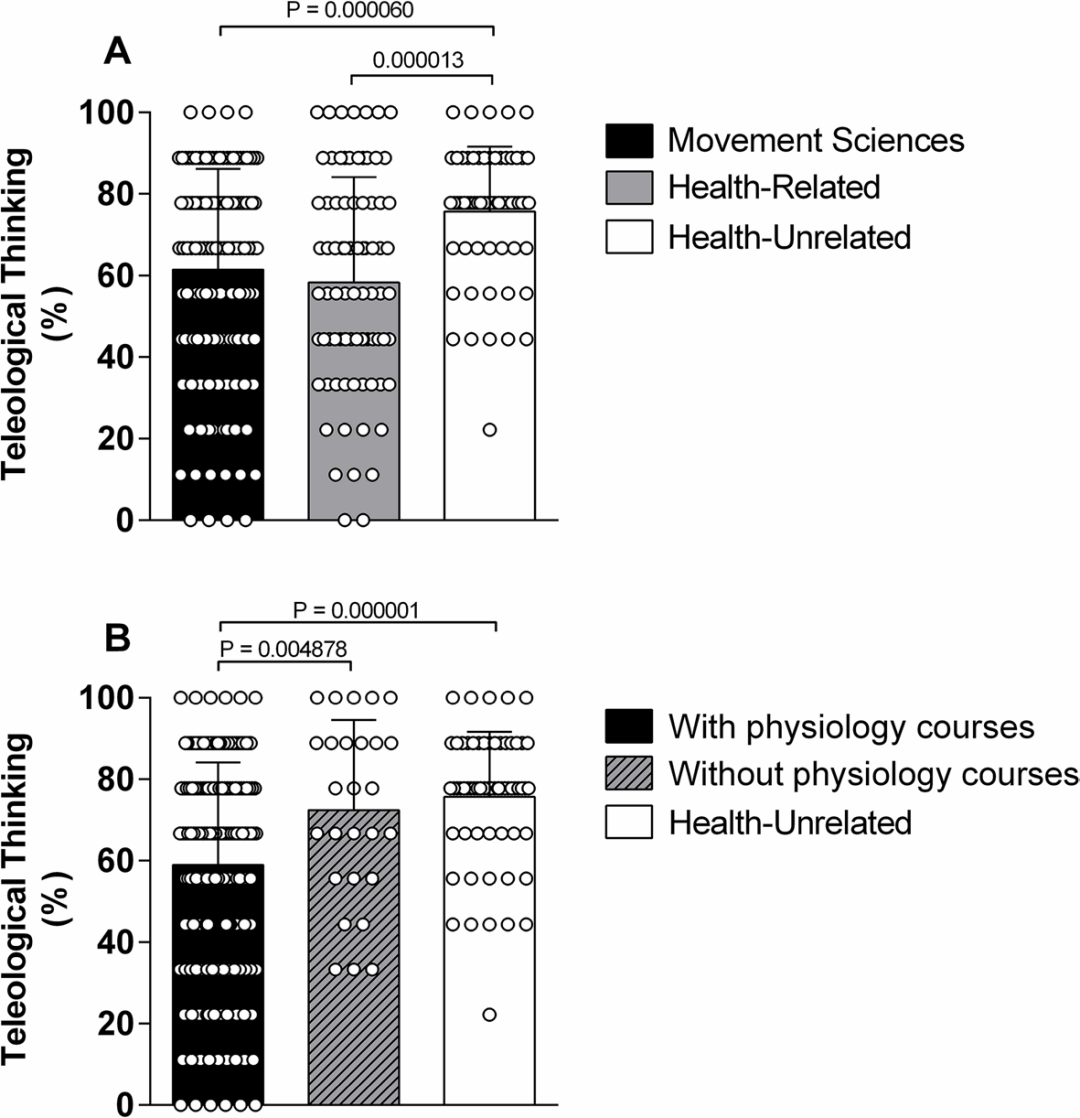
**A** Group results for teleological thinking (%) among Movement Sciences, Health-Related and Health-Unrelated courses. **B** Group results for teleological thinking (%) among students with and without physiology classes and Health-Unrelated course. Data are expressed as mean ± SD. White circles represent individual percentages. * *P* < *0.05*

Figure 1A presents the mean teleological responses among movement sciences, health-related and health-unrelated programs to test instrument. In general, groups achieved a teleological thinking of ≥ 58%. Teleological thinking of students in health-unrelated programs was significantly higher than students in health-related programs and movement sciences (76 ± 16% vs. 58 ± 26% vs. 61 ± 25%, respectively; *P* < 0.000020). There was no significant difference between movement sciences and health-related programs (*P* = 0.323867; Fig. 1A).

Figure 1B presents mean teleological thinking among those students with and without prior enrollment in physiology courses (in both movement sciences and health-related programs) and those from health-unrelated programs. There was a significant effect of previous enrollment in physiology courses. A lower percentage of teleological thinking was observed among students with prior enrollment in physiology courses compared to students without prior enrollment in physiology courses and those from health-unrelated programs (59 ± 25% vs. 72 ± 22% vs. 76 ± 16%, respectively; *P* = 0.000001, Fig. 1B). However, it is important to note that the percentage of teleological thinking even among students with prior enrollment in physiology classes remained predominant. Noteworthy, when the percentage of teleological thinking was natural logarithm-transformed for inferential analyses the results remained the same.

## Discussion

This study investigated whether undergraduate students' perception of cardiorespiratory physiology during exercise followed a predominant teleological or mechanistic thinking, and whether this proclivity was influenced by the previous enrolment in physiology courses. A salient finding was that all groups presented a predominantly (> 50%) teleological way of thinking above 58%. The teleological thinking was significantly higher in students from health-unrelated programs compared to both students in health-related programs and movement sciences. Further, although students with prior enrollment in physiology classes presented a significantly lower percentage of teleological thinking than students without physiology classes and those of health-unrelated programs, the teleological thinking remained predominant (~ 60%) highlighting that prior enrollment in physiology courses has minimal influence on the predominance of teleological vs. mechanistic thinking. Additionally, the predominance of teleological reasoning seems to be independent of the type of physiology courses’ (exercise or general physiology courses; Fig. 1). Collectively, these findings confirm the hypothesis that undergraduate students have a predominant teleological reasoning about exercise physiology, and prior enrollment in physiology courses seems not the main factor in the teleological way of thinking.

Several studies about students’ perceptions of the functioning of the human body have provided some important insights for teaching and learning physiology [9–11]. The pioneering work of Richardson [9] demonstrated, for the first time, the teleological bias in physiology in high school and undergraduate students, generating interest among researchers to further investigate the students' perception about physiology. In the present study, the percentage of teleological reasoning among students with prior enrollment in physiology courses was quite similar to those from Richardson’s study (61 vs. 59.5%, for Richardson and the present study, respectively). Overall, it is a considerably high percentage for a scientific discipline. With regards to our second hypothesis, prior enrollment in physiology courses had no meaningful influence on the predominance of the students' teleological way of thinking about physiology (75% vs. 59.5% for students without physiology and with physiology courses, respectively). The explanation for this is partially supported by previous findings demonstrating that there are no differences in the predominance of teleological thinking about physiology between high school biology students and students taking an advanced-level physiology course [9]. Alternatively, an explicit instruction approach (e.g., a lecture on the difference between mechanistic and teleological ways of thinking), as previously demonstrated [9], may have been particularly helpful in order to change the predominance of the teleological way of thinking among students.

The possible causes of the teleological tendency to attribute functions to biological phenomena have been discussed. According to Vosniadou [13], naive framework theories about natural phenomena are built in childhood and can be constantly enriched, but when it requires the revision of the presuppositions and beliefs (i.e., scientific evidence), misconceptions are most likely to occur. In contrast, Di Sessa [14] characterizes “phenomenological primitives” as small and fragmented ideas composed by abstractions of daily events, and serves to reason about novel situations and develop common sense explanations. In this perspective of cognition model, misconceptions are described as early conceptions of natural phenomena that need no further explanation. These framework theories would explain why students in this study still demonstrate teleological reasoning. Furthermore, Kelemen [7, 8] demonstrated that adults and children have a teleological interpretation of biological properties, but children also attribute purpose to all kind of objects, suggesting as a possible explanation that teleology is an innate way of thinking that becomes more selective in adulthood. Kelemen [7, 8] also considers that teleological reasoning can derive from children's understanding of intentionality and initially not be restricted to any particular phenomena. Although beyond the scope of this study, we believe that using an approach in which instructors differentiate the teleological from the mechanistic reasoning in class can result in a more adequate and complete understanding of physiology. Richardson [9] partially corroborated with this idea demonstrating that an intervention involving explanations about teleology and mechanistic in physiology was able to considerably decrease students' teleological reasoning. Unfortunately, given the amount of subjects and programs enrolled in the present investigation we were unable to obtain the information on the physiology content, teaching pedagogies, assessment methods and learning outcomes. Therefore, we cannot determine how different teaching approaches could have affected our main findings and thus it should be considered as a limitation.

Physiology is perceived by students and teachers as a challenging discipline, where students' difficulties are not attributed to instruction, but rather to the discipline's inherent difficulty [1, 15, 16]. On the other hand, students and instructors attribute learning difficulties to causal and teleological reasoning [1, 16], which could probably be avoided with appropriate instruction in terms of cause and effect. Modell et. al. [17] defend that instructors must detect and expose students’ misconceptions and offer the tools to conceptual change in support of scientific knowledge. It is equally important that students actively participate in the learning process by recognizing that their comprehension is incorrect and building a new mental model [18]. Additionally, Cliff et. al. [19] have shown that case study analysis provided a 36% remediation of students' misconceptions about respiratory physiology, suggesting that a learning environment where students actively confront their understanding of the physiological phenomena is useful to help avoid or correct misconceptions. The approach to the learning processes, like deep learning, strong pedagogical strategies, and well-designed structured curriculum and lessons are desirable from the instructor and could influence students towards a more mechanistic thinking [19]. However, this learning process requires active participation, construction and reconstruction, integration and reintegration of cognitive and action structures, a process that requires sustained effort [20].

One way to improve this is through the fact that students appreciate some disciplines components with practical applicability. Extended practical projects could also provide an increase in student interest and motivation during the learning process [21]. For example, the incorporation of active learning activities in conjunction with more traditional approaches to teaching in the classroom has proven to be more effective for student learning and retention compared with lecture alone [22, 23]. With this in mind, Teixeira et. al. [23] described a practical physiology laboratory class using isolated skeletal muscle metaboreflex activation to teach cardiovascular physiology for undergraduate students. This approach was able to significantly improve students’ level of understanding regarding several cardiovascular responses to exercise, reinforcing their appreciation for the importance of the subject matter and enhancing their desire to learn. Therefore, we expect that practical demonstrations of how physiological phenomena happen would potentially avoid misconceptions about physiological events and consequently the teleological thinking in physiology courses. Future studies are needed to explore whether teaching strategies could have influence on teleological/mechanistic thinking in physiology courses.

It is important to consider some of the potential limitations of the present study. One could argue that students might choose the teleological answers in our test instrument simply because they have never even seen many of the words in the mechanistic answer. However, whether students in health-related courses chose the teleological answers simply because they have never even seen many of the words in the mechanistic answer, would reinforce that the teaching process is based on consequences (teleological) rather than the cause (mechanistic). Noteworthy, the test instrument used the same structure from Richardson´s paper [9], but with reference to cardiorespiratory responses to exercise. All questions were based on typical physiological responses to exercise and/or muscle contraction. Also, it is important to note that extraneous factors could have impacted students’ choices. For example, access time to the questionnaire was not limited and students may have consulted external sources (e.g., internet, books, or another student). Another factor to be considered is that the curricula, access to patients/clients, age and year of college, and pedagogical language (i.e., teaching/textbooks), may have impacted students’ responses to the test instrument. Lastly, the test instrument only gave students one chance to select an option and has only one tier, which limit the fully understanding of students' reasoning and can lead to potential guessing.

In summary, this study confirmed the hypothesis that undergraduate students have a tendency to choose teleological as opposed to mechanistic thinking in exercise physiology. Further, prior enrollment in physiology courses has a minimal influence on the predominance of teleological reasoning among undergraduate students, suggesting that teleological thinking is partially independent of the degree of familiarity with this discipline.

## Data Availability

The datasets used and/or analysed during the current study are available from the corresponding author on reasonable request.
